# Cross-sectional evaluation of the saccharin transit time test for primary ciliary dyskinesia: did we discard this tool too soon?

**DOI:** 10.1590/1516-3180.2022.0508.R2.13032023

**Published:** 2023-05-12

**Authors:** Mariana Dalbo Contrera Toro, Erica Ortiz, Fernando Augusto Lima Marson, Laíza Mohana Pinheiro, Adyléia Aparecida Dalbo Contrera Toro, José Dirceu Ribeiro, Eulália Sakano

**Affiliations:** I MD, MSc. PhD Student, Department of Otolaryngology, Faculty of Medical Sciences, Universidade Estadual de Campinas (UNICAMP), Campinas (SP), Brazil.; II MD, PhD. Physician, Department of Otolaryngology, Faculty of Medical Sciences, Universidade Estadual de Campinas (UNICAMP), Campinas (SP), Brazil.; III MD, PhD. Physician, Department of Pediatrics, Faculty of Medical Sciences, Universidade Estadual de Campinas (UNICAMP), Campinas (SP), Brazil; Researcher, Laboratory of Medical Genetics and Genome Medicine, Department of Medical Genetics, Faculty of Medical Sciences, UNICAMP, Campinas (SP), Brazil; Professor, Laboratory of Human and Medical Genetics, Universidade São Francisco, Bragança Paulista (SP), Brazil.; Universidade Estadual de Campinas, Faculty of Medical Sciences, Laboratory of Medical Genetics, Campinas, SP, Brazil; Universidade São Francisco, Laboratory of Human and Medical Genetics, Campinas, SP, Brazil; IV MD. Physician, Department of Otolaryngology, Faculty of Medical Sciences, Universidade Estadual de Campinas (UNICAMP), Campinas (SP), Brazil.; V MD, PhD. Adjunct Professor, Department of Pediatrics, Faculty of Medical Sciences, Universidade Estadual de Campinas (UNICAMP), Campinas (SP), Brazil.; VI MD, PhD. Pediatric Pulmonologist and Full Professor, Department of Pediatrics, Faculty of Medical Sciences, Universidade Estadual de Campinas (UNICAMP), Campinas (SP), Brazil.; VII MD, PhD. Adjunct Professor, Department of Otolaryngology, Faculty of Medical Sciences, Universidade Estadual de Campinas (UNICAMP), Campinas (SP), Brazil.

**Keywords:** Ciliary motility disorders, Microscopy, electron, transmission, Nasal mucosa, Saccharin, Bronchiectasis, Clinical diagnosis, Ciliary function, Chronic rhinosinusitis

## Abstract

**BACKGROUND:**

Primary ciliary dyskinesia (PCD) is a rare and heterogeneous disease that is difficult to diagnose and requires complex and expensive diagnostic tools. The saccharin transit time test is a simple and inexpensive tool that may assist in screening patients with PCD.

**OBJECTIVES:**

This study aimed to compare changes in the electron microscopy findings with clinical variables and saccharin tests in individuals diagnosed with clinical PCD (cPCD) and a control group.

**DESIGN AND SETTING:**

An observational cross-sectional study was conducted in an otorhinolaryngology outpatient clinic from August 2012 to April 2021.

**METHOD:**

Patients with cPCD underwent clinical screening questionnaires, nasal endoscopy, the saccharin transit time test, and nasal biopsy for transmission electron microscopy.

**RESULTS:**

Thirty-four patients with cPCD were evaluated. The most prevalent clinical comorbidities in the cPCD group were recurrent pneumonia, bronchiectasis, and chronic rhinosinusitis. Electron microscopy confirmed the clinical diagnosis of PCD in 16 of the 34 (47.1%) patients.

**CONCLUSION:**

The saccharin test could assist in screening patients with PCD due to its association with clinical alterations related to PCD.

## INTRODUCTION

Primary ciliary dyskinesia (PCD) is an autosomal recessive disease in which ciliary motility is compromised by mucus accumulation, changes in the microbiota of the airways, infection, structural changes with consequent functional worsening, and important clinical repercussions.^1,2^

The clinical changes secondary to ciliary motility dysfunction include defects in laterality (situs inversus, situs inversus totalis, and dextrocardia), infertility, chronic rhinosinusitis (CRS), chronic otitis media, and recurrent infections of the upper and lower airways.^1–3^ In the lungs, changes in mucociliary clearance are related to respiratory failure in the neonatal period, recurrent pneumonia, bronchiectasis, and chronic cough.^1,3–5^

There is no gold standard for PCD diagnosis.^1,2^ It is a rare disease that affects approximately 1:10,000 live births.^1^ Furthermore, it is a constantly evolving condition in terms of diagnosis, and some PCD phenotypes are not yet fully established.^1,2^

Currently, guidelines from the American Thoracic Society (ATS) and the European Respiratory Society (ERS) suggest diagnostic confirmation through a combination of different clinical suspicion and diagnostic methods such as nasal nitric oxide (nNO), transmission electron microscopy (TEM), high-speed video microscopy, and genetic screening for pathogenic variants in PCD-related genes.^1,6^ Clinical scores such as the Primary Ciliary Dyskinesia Rule (PICADAR) and ATS clinical screening questionnaires can help in diagnosing this disorder.^1,6,7^

TEM involves the analysis of ciliated epithelium samples. In this analysis, alterations in the ciliary ultrastructure were evaluated, and approximately 70% of patients with PCD presented with alterations within the TEM.^8^ Some patients with PCD may not present obvious defects under TEM, even with changes in ciliary function.^2,8^

Infectious and inflammatory processes can affect mucociliary transport, so false positives can be found in these cases.^9^ On the other hand, patients with normal ciliary ultrastructure may present functional defects of ciliary motility.^8^

The saccharin test allows for a rough evaluation of mucociliary function. It is a screening test that is widely available outside specialized centers; it is simple, inexpensive, easy to implement, and can be a useful tool for general practitioners.^10–12^ Nevertheless, it is a subjective test that may be normal in patients with dyskinetic beating cilia and difficult to perform in children younger than 12 years.^12^

Adequate diagnostic evaluation remains a challenge for the management and follow-up of patients with PCD.^1,2,13,14^ There have been many changes in the diagnostic criteria and methods since the prior studies on the use of saccharin transit time tests were conducted.^1,2^ Therefore, this study is justified, as it could verify the saccharin test as a screening prospect.

## OBJECTIVE

This study aimed to compare changes in TEM, clinical variables, and the saccharin transit time in individuals with a clinical PCD diagnosis.

## METHODS

This cross-sectional prospective observational study included patients from the Hospital de Clínicas of the Universidade Estadual de Campinas (HC-UNICAMP). Before beginning the study, all participants and their guardians signed an informed consent form. The study was approved by the ethics committee of UNICAMP (CAAE:1109.0.146.000-11 approved on January 17, 2011, and CAAE: #31498020.8.0000.5404 approved on June 4, 2020).

Patients with a clinical PCD (cPCD) diagnosis based on otorhinolaryngology, pneumopediatrics, and pulmonology between August 2012 and April 2021 were included in the cPCD group. The clinical diagnosis was based on the characteristic symptoms described by the ERS task force criteria: defects of laterality, family history of PCD, persistent rhinorrhea, CRS, neonatal respiratory failure, productive cough, bronchiectasis, chronic otitis (chronic otitis media, serous otitis media, conductive hearing loss), and infertility.^1^

Patients diagnosed with cystic fibrosis, alpha-1-antitrypsin deficiency, or immunodeficiencies, and smokers were excluded. Patients with insufficient material for TEM were excluded. Patients who presented with acute upper airway infections on the day of the appointment were rescheduled.

Because PCD is a rare disease, the sample size was based on a convenience sample of patients who agreed to participate in the study.

All patients answered a clinical form containing demographic data, characteristic PCD symptoms, and personal history, and those evaluated after 2016 answered the PICADAR and ATS clinical screening questionnaires.^6,7^

All patients underwent nasal endoscopy, and the main findings were documented. This examination allowed the nasal fossa to be biopsied and tested, excluding obstructive factors.

The saccharin test was performed as described previously.^10,11,15^ A sodic saccharin fragment measuring 1 mm in diameter was placed on the surface of the head of the inferior nasal turbinate 1 cm posterior to the nasal vestibule to avoid the squamous epithelium area. The participants remained seated, breathing normally, without sneezing or blowing their nose. The time between the placement of saccharin and the beginning of the sensation of sweet taste was measured in minutes. If the patient did not report a sensation of taste after 60 minutes, the test was interrupted. The test was considered altered when the result was greater than 30 minutes.^11^

For the TEM evaluation, the material was collected through cytological brushing of the inferior turbinate. The material was placed in as container with a glutaraldehyde fixing solution of 3% and kept at 4 °C for three hours. The biopsy specimens were processed, washed, and placed in a phosphate buffer container. The samples were analyzed by two researchers (MDCT and EO) according to the international consensus guideline for reporting TEM (BEAT PCD TEM criteria).^16^ Changes in the ultrastructure were based on the observation of at least 100 cilia, being evaluated in cross-sections.^1^ Abnormalities found in less than 10% of the cilia were considered within the normal range.^17^ Described alterations associated with PCD were analyzed, such as the absence of the internal and external arm of dynein, translocations, and absences of central microtubules, compound cilia, ciliary disorientation, and alterations of peripheral and central microtubules.^18,19^

The BEAT PCD TEM criteria consist of class 1 alterations: hallmark defects such as more than 50% of axonemes with outer dynein arm (ODA) defects with or without inner dynein arm (IDA) defects or microtubular disorganization (MD) with IDA defects, and class 2 alterations: cilia alterations that confirm PCD diagnosis in the presence of other supporting evidence which include: central complex defects, mislocalization of basal bodies with few or no cilia (Oligocilia), MD defect with IDA present or ODA defect with or without IDA defect in 25-50% of cross-sections.^16^

Descriptive analyses were performed using categorical data and absolute and relative frequencies. Numerical data are presented as the median, minimum, and maximum values and interquartile intervals. The normality of the numerical data was evaluated using the following techniques: (i) analysis of descriptive measures for central tendency and (ii) statistical tests (normality tests): Kolmogorov-Smirnov and Shapiro-Wilk. The data collected from the biopsies were compared between the groups using statistical analysis of contingency (chi-square), Fisher’s exact test, and the Wilcoxon-Mann-Whitney test. The significance level was set at P < 0.05.

## RESULTS

**Figure 1:**
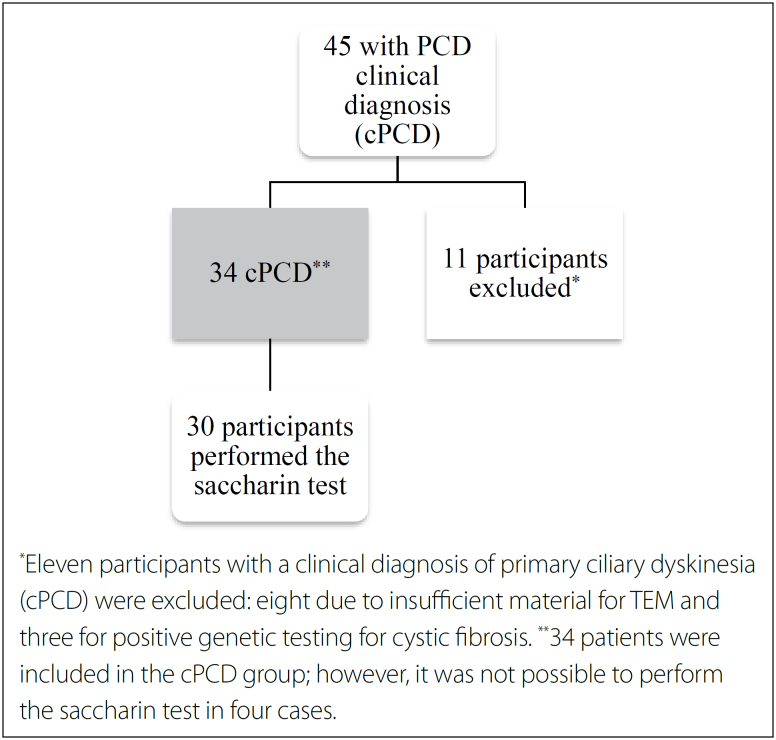
Flow chart of the inclusion of participants in the study.

A total of 45 individuals were evaluated. Eight patients with cPCD were excluded because of insufficient material for TEM. Three patients were excluded after testing positive for the cystic fibrosis transmembrane conductance regulator gene. After exclusion, 34 patients were included in this study. Moreover, four patients were unable to complete the saccharin transit time test because of a lack of understanding or reactive sneezing during the test. Figure 1 shows a flowchart of the inclusion of patients, and Table 1 shows the main clinical characteristics of the study participants. The median age of the participants was 15.5 years (range: three to 60 years). The most common clinical features were bronchiectasis, recurrent pneumonia, and CRS.

**Table 1:** Main characteristics of participants with a clinical diagnosis of primary ciliary dyskinesia (cPCD)

	cPCD group n = 34
Median age	15.5
Sex: male	22 (64.7%)
Recurrent pneumonia	23 (67.6%)
Laterality defects	9 (26.5%)
Fertility disorders	4 (11.8%)
Chronic rhinosinusitis	19 (55.9%)
Chronic otitis media	13 (38.2%)
Bronchiectasis	26 (76.5%)
Asthma	15 (44.1%)
Allergic rhinitis	17 (50%)
Saccharin transit time > 30 minutes^*^	7 (23.3%)

* In the cPCD group, only 30 patients were able to complete the saccharin transit time test.

**Figure 2:**
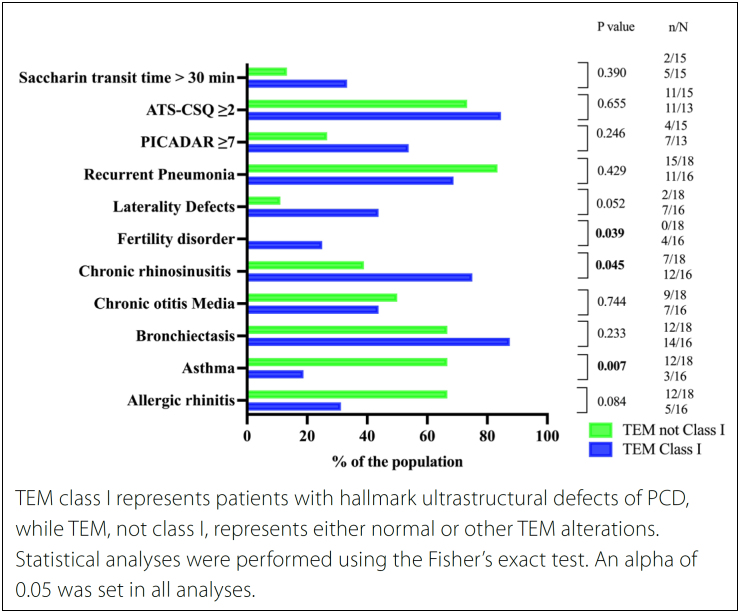
Clinical characteristics, clinical scores of PICADAR and ATS-CSQ (American Thoracic Society clinical screening questionnaire), and alteration in the saccharin progression time test (> 30 min) of individuals clinically classified with primary ciliary dyskinesia (cPCD), divided by the transmission electron microscopy (TEM) findings.

Twenty-seven (79.4%) individuals presented with changes in ciliary ultrastructure, and seven (20.6%) had no alterations on TEM. When classifying these alterations according to the BEAT PCD TEM criteria,^16^ 16 patients (47.1%) presented class I alterations, five patients (14.7%) presented class II alterations, and 13 patients (38.2%) did not present alterations compatible with the PCD diagnosis. Figure 2 shows the clinical alterations, clinical scores of the PICADAR and ATS-CSQ, and saccharin transit time in the groups with or without class I alterations.

Patients with cPCD present with the following alterations in ciliary ultrastructure: absence of dynein’s inner arm, absence of dynein’s external arm, ciliary disorientation, compound cilia, central microtubule translocation, extra peripheral microtubules, extra central microtubules, absence of cilia, absence of peripheral microtubules, and absence of central microtubules.

**Figure 3:**
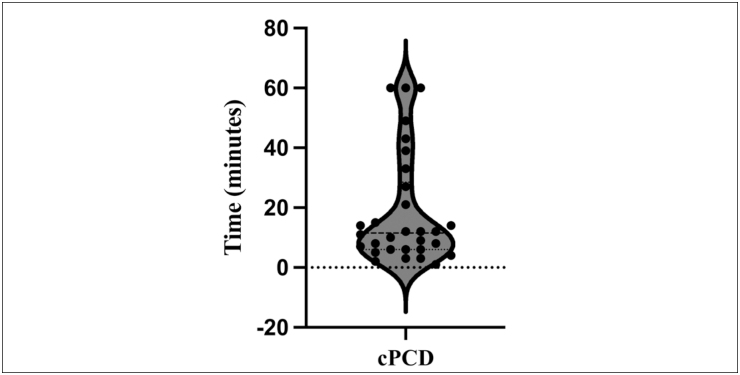
Violin plot for the saccharin progression time (minutes) of individuals clinically classified with primary ciliary dyskinesia (cPCD).

The median saccharin progression time was 11.5 minutes. Figure 3 shows the distribution of the results for the participants who completed the saccharin transit time test. No association was found between the altered saccharin test results and changes in TEM in general or according to the BEAT PCD TEM criteria.

No association was found between PICADAR ≥ 7 or the ATS clinical score ≥ 2 and saccharin test greater than 30 minutes. Also, there was no association between PICADAR ≥ 7 or the ATS clinical score ≥ 2 and TEM class I defects.

## DISCUSSION

The diagnosis of PCD remains a major challenge in clinical practice due to the need for a combination of tools, which often require sophisticated techniques available in only a few centers in a limited number of countries.^13^ The investigation of this disease becomes even more difficult due to the low incidence and great variability of genotypes and phenotypes.^7^ In Brazil, few studies have been conducted that evaluate the diagnosis and clinical characteristics of patients with PCD.^20^

Due to this phenotypic unpredictability, great heterogeneity may be observed in the clinical characteristics of these patients. In this study, clinical variability was observed in the cPCD group, and the most frequent features were recurrent pneumonia, bronchiectasis, and CRS.

Identifying children with suspected PCD at an early age can improve the prognosis and delay pulmonary remodeling, leading to a decrease in pulmonary function.^6,21^ However, complex and expensive screening and diagnostic tests may delay PCD diagnosis. Pediatric European centers demonstrated a 5.3-year median age at diagnosis.^21^ However, Braun et al. demonstrated a median age of 17 years at PCD diagnosis in a retrospective 30-year analysis of a single center,^22^ similar to the results of this study.

Three meta-analyses of studies with patients with PCD showed differences in the prevalence of clinical features such as CRS, bronchiectasis, situs inversus, otitis media, and recurrent pneumonia. There is great heterogeneity in the prevalence of clinical characteristics.^5,23–25^

Seventy-seven percent of the patients with clinical characteristics compatible with PCD presented with alterations in the ciliary ultrastructure on TEM analysis. This result was consistent with a meta-analysis, which reported a detection rate of 83%.^25^ In the literature, approximately 30% of PCD cases present normal TEM.^8^

The findings of this study concerning TEM were similar to those of a study by Demarco et al. in the Brazilian population, which showed 54% alterations in dynein arms (internal, external, or associated) and 14% ciliary aplasia.^9^

However, when analyzing the criteria published by the International Consensus of TEM (BEAT PCD TEM criteria) in 2020, 61.8% of our patients were classified as having either class I or II TEM alterations.^16^

Previously, the saccharin test was disregarded in the new diagnostic guidelines of 2017 and 2018 because of its technical difficulty, especially in children.^1,26^ A previous study demonstrated a sensitivity of up to 95% in identifying a normal ciliary ultrastructure.^17^ However, our results showed no relationship between the altered saccharin test and TEM. Patients with ciliary beat alterations may also present false negatives, which may be true in other diagnostic tests, such as TEM and genetic screening.^11,27^

Although several guidelines state that the saccharin transit time test is unreliable in children younger than 12 years old,^11^ studies have shown reliable results when testing patients aged three to 11 years old for other conditions such as adenoid hypertrophy and even healthy children.^27–29^

Our study found no association between altered clinical scores, such as the PICADAR and ATS-CSQ, and alterations in the saccharin test or TEM. Clinical scores have gained great relevance in the diagnostic algorithm, especially in the ATS guidelines, where patients with a clinical screening questionnaire score of less than two should not continue the investigation.^2^ The positive predictive value of these scores in previous studies was similar to that of nNO, but these scores require multicentric and multidisciplinary validation.^13^

The complete diagnostic algorithm for PCD can cost €653 to €2,097 per patient, which can be challenging in countries with limited resources and social heterogeneity, such as Brazil. This is not only due to the costs but also the lack of reference centers with staff able to perform the required tests and analyses.^26,30^

Thus, physicians should pay attention to patients with severe or atypical symptoms and individually evaluate each patient’s clinical history.^1^ In this context, the assessment of the saccharin transit time may be an additional tool to corroborate subjective clinical decisions, particularly in primary and secondary care centers.

Our study has some limitations because it examined a rare disease and reduced the number of patients evaluated per year. In the nine years of analysis included in this study, there were changes in the diagnostic criteria, especially concerning the TEM criteria, and scores such as the PICADAR and ATS clinical questionnaire were incorporated before 2016. In addition, evaluating other tools is challenging because of the lack of a reference test for PCD diagnosis. Access to nNO, ciliary beat analysis through video microscopy, and genetic testing may be useful in future studies that diagnostic and screening tools.

## CONCLUSION

Due to the genotypic and phenotypic complexity of PCD, this study showed that the saccharin transit time test and TEM may be complementary to other more specific tools. Nevertheless, the saccharin transit time cannot be used as a diagnostic test because of its lack of association with TEM alterations.
